# Heading Estimation of Robot Combine Harvesters during Turning Maneuveres

**DOI:** 10.3390/s18051390

**Published:** 2018-05-01

**Authors:** Md Mostafizar Rahman, Kazunobu Ishii

**Affiliations:** 1 Graduate School of Agriculture, Hokkaido University, Kita-9, Nishi-9, Kita-ku, Sapporo 060-8589, Japan; mmr.fet@gmail.com; 2 Department of Food Engineering and Tea Technology, Shahjalal University of Science and Technology, Sylhet 3114, Bangladesh; 3 Research Faculty of Agriculture, Hokkaido University, Kita-9, Nishi-9, Kita-ku, Sapporo 060-8589, Japan

**Keywords:** tracked combine harvester, dynamic model, extended Kalman filter, global positioning system, inertial measurement unit, crop periphery

## Abstract

Absolute heading is an important parameter for a robot combine harvester or a robot tracked combine harvester, especially while it is turning, but due to the rapid turning of robot combine harvesters, its inertial measurement unit gives a gyro measurement bias that causes heading drift. Our research goal is to estimate the absolute heading of robot combine harvesters by compensating this gyro measurement bias during non-linear turning maneuvers. A sensor fusion method like the extended Kalman filter combined with the tracked combine harvester dynamic model and sensor measurements was used to estimate the absolute heading of a robot combine harvester. Circular, sinusoidal and concave shapes were used to evaluate the estimated heading produced by the sensor fusion method. The results indicate that the estimated heading is better than measured heading which was calculated from the integration of yaw rate gyro measurements, and the root mean square errors (RMSEs) for estimated headings are smaller than the measured headings. In practics, the target of this paper is thus the estimation of a heading or absolute heading that is bias compensated, and can be further used to calculate the exact crop periphery for automatic path planning of robot combine harvesters.

## 1. Introduction

Unlike wheeled vehicles, tracked vehicles are widely popular due to their non-linear contact characteristics between the tracks and the ground, which allows them to be operated under adverse field conditions for agricultural production, and allows turning at high speed with a small turning radius or higher steering command. Under autonomous conditions, the tracked vehicle has a global positioning system and inertial sensor for providing the vehicle state and direction, but when this tracked vehicle is turning, the inertial sensor reading has measurement uncertainties. Since the vehicle direction is more important for autonomous guidance and other navigation purposes, it is necessary to compensate this uncertainty of the inertial sensor measurements. 

The main controlling feature of auto guidance is to steer the vehicle to follow a desired path automatically, which requires a proper guidance system able to detect the vehicle’s position and direction, create proper steering signals, and steer the vehicle according to the signals [1].There are different guidance sensing systems, including global positioning system, inertial sensors, geomagnetic direction sensors, machine vison and laser scanners which are used to find the control parameters of the autonomous vehicles such as heading and offset [2,3,4,5]. The functional characteristics of each sensor provides the desired information, which contains erroneous measurement readings due to noise, measurement errors, and time delays. In general, a single sensor is not able to provide enough information, whereas multi-sensor integration can provide more useful information, which is more helpful and informative than what can be observed using a single sensor. This information needs to be fused in a way that reduces sensor uncertainties and the additional task of interpretation must be performed [6]. There are different approaches used for sensor fusion to obtain the position and direction of vehicles, such as complementary filters [7] or Kalman filters with various architectures [8,9,10,11,12,13], particle filters or sequential Monte Carlo methods [14,15,16,17,18]. Since the dynamic motion of a vehicle is non-linear, a non-linear dynamic model and extended Kalman filter are commonly used for navigation purposes. For instances, Rohac [19] addressed a cost-effective solution by using a non-linear observer and extended Kalman filter with commercial grade inertial sensors, and studied the performances of different approaches to obtain navigation solutions with robustness to GNSS outages. Noguchi [20] developed a guidance system based on the real time global positioning system (RTK-GPS), geomagnetic sensor (GDS) and machine vision by using an extended Kalman filter method which provided the most appropriate vehicle heading in real time. Alatise and Hancke [21] used an extended Kalman filter with inertial sensors and camera to estimate the position and direction of a mobile robot. With a non-linear extended Kalman filter, a tracked vehicle model and sensor measurements are used to estimate the trajectory, orientation and other soil parameters for small-scale tracked vehicles [22]. 

**Figure 1:**
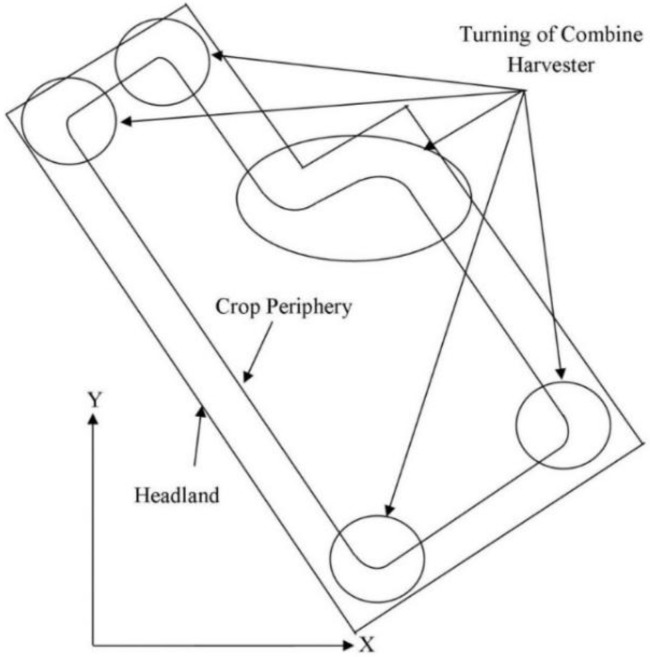
A polygonal field with a circle representing the turning area of the tracked combine harvester.

An autonomous tracked combine harvester developed by Zhang [23] was used to cut wheat and paddy rice in real time by using an appropriate harvesting map which is created based on a real time global positioning system (RTK-GPS) and inertial measurement units (IMUs); before harvesting, the outside crop near to headland is cut twice or thrice by the tracked combine harvester, and during this time the sensor measurements are logged for making a navigation map. Generally, the tracked combine harvester makes turns at moderate to high speed with a small turning radius, which is very popular with farmers, and this turning position is represented by a circle marked in Figure 1. During this turn, the measured heading of a tracked combine harvester contains drift errors, which result from the IMU gyro measurement bias. To get an absolute heading, this bias needs to be compensated by using a tracked combine harvester model and sensor fusion method. In this research, the main contributions are the estimated absolute heading of tracked combine harvester during non-linear conditions (especially high speed turns with small turning radii) by using a tracked combine harvester dynamic model which was developed by us based on a real time global positioning system (RTK-GPS) and inertial measurement units (IMU). For more details readers should see [24]. In practice, this estimated heading can further be used to obtain the exact crop periphery for calculating the harvesting map of a robot combine harvester (for more details see [25]).

## 2. Materials and Methods

### 2.1. System Platform and Sensors

**Figure 2:**
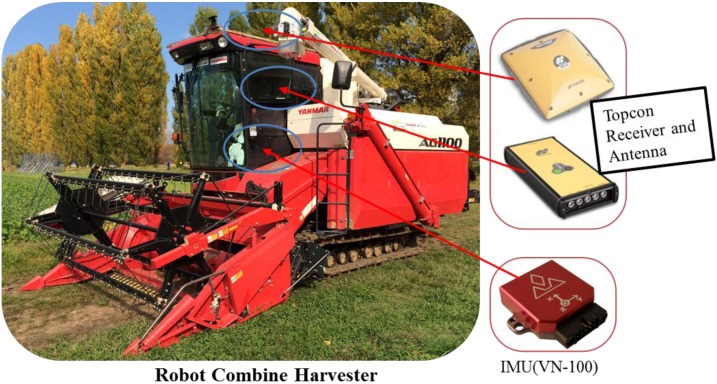
View of the robot combine harvester equipped with RTK-GPS and IMU sensors.

A model AG1100 tracked combine harvester (Yanmar Co., Ltd, Japan) was used as a prime research platform, which was equipped with a Real Time Kinematic Global Positioning System (RTK-GPS) and Inertial Measurement Unit (IMU) as shown in Figure 2. This tracked combine harvester is converted into a robot combine harvester which is fully controlled by a CAN bus, and the speed of the tracked combine harvester ranges from −2.0~2.0 m/s, approximately. This harvester can be used to harvest cereal crops like paddy rice and wheat. The VN-100 IMU (VectorNav Technologies, Dallas, Texas, USA) is a miniature, high performance Inertial Measurement Unit and Attitude Heading Reference System (AHRS) which is used to measure the heading and angular rate of the tracked combine harvester. Its dynamic accuracy for the yaw rate is 1.0° RMS, heading’s dynamic accuracy: 2.0° RMS and the gyro has an alignment error: ±0.05°, noise density: <0.0035°/s/√Hz, in-run bias stability: <10°/h. This IMU, consisting of three accelerometers, three magnetometers and three rate gyros, provides nine axes, which are sampled at a rate of 200 Hz through a USB serial port. On the other hand, the update rate of the RTK-GPS is up to 20 Hz, although for logging of the RTK-GPS output, the update rate is maintained at 5 Hz or 10 Hz and the Baud rate was 115,200, where the IMU also matches that update rate. The RTK correction signal was calculated from a Virtual Reference Station (VRS) via an Internet connected to the on-board computer that logs the data from the RTK-GPS receiver through an RS232C serial port. These sensor measurements were logged into the control PC of the tracked combine harvester as a GGA header for RTK-GPS and VNIMU header for IMU. The GGA header from the RTK-GPS sensor gives the position of the tracked combine harvester, whereas the VNIMU header provides bias-uncompensated IMU measurements such as magnetic, acceleration and angular rate measurements, temperature and pressure. In this research, only position and bias uncompensated angular rate data were considered to estimate the absolute heading of robot combine harvester under non-linear conditions.

### 2.2. Tracked Combine Harvester Model

**Figure 3:**
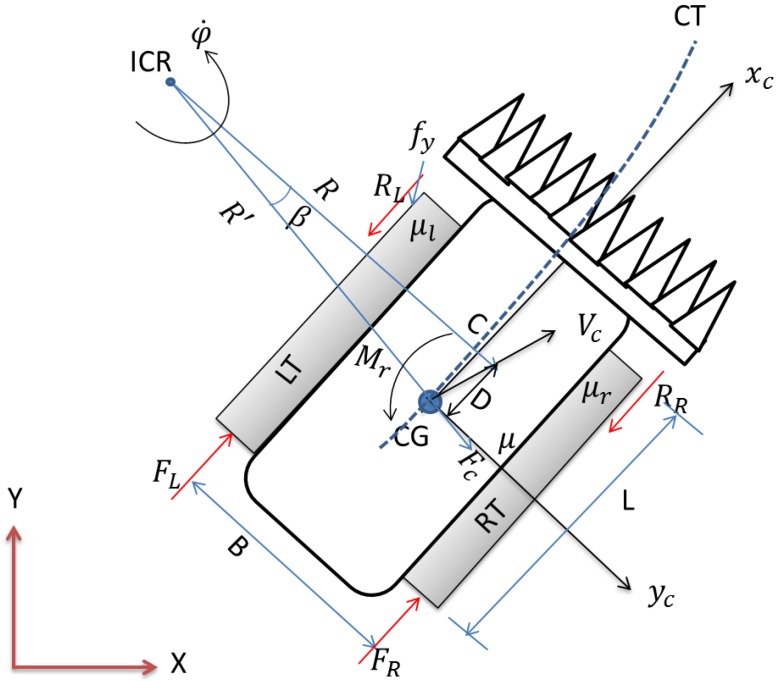
Free body diagram of the tracked combine harvester dynamic model.

Figure 3 shows the free body diagram of dynamic model for the tracked combine harvester moving on a general plane, turning to the left or counter-clockwise [24,26,27]. Its acceleration is in the positive $x_{c},\;y_{c}$ and φ directions. The external thrusts and resistive forces acting on the tracked combine harvester are$\;F_{R}$, $F_{L}$ and $R_{R}$, $R_{L}$, respectively. The value $f_{y}$ indicates the lateral friction force due to the effect of lateral soil shear. Figure 3 is represented in the global reference frame XYZ, which indicates the tracked combine harvester turns around an instantaneous center of rotation (ICR). The angle β is called sideslip angle and is determined from the velocity $V_{c}$ and the longitudinal axis $x_{c}$ of the tracked combine harvester. It is assumed that the normal pressure distribution along the track is non-uniform, and the coefficient of lateral resistance μ is not constant. The instantaneous center of rotation must shift forwards of the tracked combine harvester centroid by the amount of D, as shown in Figure 3. This longitudinal shifting D depends on the tracked combine harvester lateral acceleration [27]. D is required to develop a net lateral force that accelerates the tracked combine harvester towards the instantaneous center of rotation, and also minimizes the resistive yawing moment [28]. The centrifugal force $F_{c}$ acting on the tracked combine harvester is given in Figure 3. We also described this tracked combine harvester model based on sensor measurements so that it can be further used for autonomous navigation purposes [24].

For a tracked combine harvester of mass m and a moment of inertia about the center of mass I, the equations of motion can be written in the body reference frame by using Equations (1)–(3), respectively:

(1) $$m{\ddot{x}}_{c}=F_{R}+F_{L}-R_{R}-R_{L}-F_{c}\sin\beta$$

(2) $$m{\ddot{y}}_{c}=F_{c}\cos\beta -\mu mg$$

(3) $$I\ddot{\varphi }=\frac{\left[\left(F_{R}-R_{R}\right)-\left(F_{L}-R_{L}\right)\right]B}{2}-M_{r}$$

Using a kinematic model of the tracked combine harvester, the velocity $V_{c}$ is calculated based on each track’s velocity including the slip of tracks from the tracked combine harvester expressed by Equation (4). The RTK-GPS positions used to calculate the actual turning radius R by using the least square method [24] is given by Equation (6), which was derived from the general circle Equation (5), where (a, b) and C are the center circle and coefficient:

(4) $$V_{c}=\frac{V_{R}\left(1-S_{r}\right)+V_{L}\;\left(1-S_{l}\right)}{2}$$

(5) $${\left(a-x\right)}^{2}+{\left(y-b\right)}^{2}=R^{2}$$

(6) $$R=\sqrt{(a^{2}}+b^{2}-C)$$

Now, Equations (1) and (2) are integrated to transfer the longitudinal and lateral direction velocities of the tracked combine harvester to the local coordinates. In order to run this tracked combine harvester in real time, the velocities in the longitudinal and lateral direction in the harvester coordinates are expressed in a global reference frame by the Equation (7):

(7) $$\left[\begin{array}{c} \dot{X}\\ \dot{Y} \end{array}\right]=\left[\begin{array}{cc} \cos\varphi & -\sin\varphi \\ \sin\varphi & \cos\varphi \end{array}\right]\left[\begin{array}{c} {\dot{x}}_{c}\\ {\dot{y}}_{c} \end{array}\right]$$

By using the Equation (7), the tracked combine harvester model equations can be rewritten as Equation (8):

(8) $$\left[\begin{array}{c} {\dot{X}}_{c}\\ {\dot{Y}}_{c}\\ \dot{\varphi } \end{array}\right]=\left[\begin{array}{c} \left(\frac{1}{m}\left(F_{R}+F_{L}-R_{R}-R_{L}-F_{c}\sin\beta \right)\cos\varphi -\frac{1}{m}\left(F_{c}\cos\beta -\mu mg\right)\sin\varphi \right)\Delta t\\ \left(\frac{1}{m}\left(F_{R}+F_{L}-R_{R}-R_{L}-F_{c}\sin\beta \right)\sin\varphi +\frac{1}{m}\left(F_{c}\cos\beta -\mu mg\right)\cos\varphi \right)\Delta t\\ \left(\frac{\left[\left(F_{R}-R_{R}\right)-\left(F_{L}-R_{L}\right)\right]B}{2}\;-M_{r}\right)\frac{\Delta t}{I} \end{array}\right]$$

The discrete-time process model is developed from the continuous-time process model, where the state space equation is obtained by integrating the continuous-time equations over the interval from $t_{k}$ to $t_{k+1}$. The discrete-time model is an approximation of the continuous-time model. Now, the discretization of the Equation (8) can be written by the following Equation (9):

(9) $$\left[\begin{array}{c} {\dot{X}}_{k+1}\\ X_{k+1}\\ \begin{array}{c} {\dot{Y}}_{k+1}\\ Y_{k+1}\\ \begin{array}{c} {\dot{\varphi }}_{k+1}\\ \varphi_{k+1} \end{array} \end{array} \end{array}\right]=\left[\begin{array}{c} {\dot{X}}_{k}+\left(\frac{1}{m}\left(F_{R}+F_{L}-R_{R}-R_{L}-F_{c}\sin\beta \right)\cos\varphi -\frac{1}{m}\left(F_{c}\cos\beta -\mu mg\right)\sin\varphi \right)\Delta t\\ X_{k}+{\dot{X}}_{k}\Delta t+\left(\frac{1}{m}\left(F_{R}+F_{L}-R_{R}-R_{L}-F_{c}\sin\beta \right)\cos\varphi -\frac{1}{m}\left(F_{c}\cos\beta -\mu mg\right)\sin\varphi \right)\frac{\Delta t^{2}}{2}\\ {\dot{Y}}_{k}+\left(\frac{1}{m}\left(F_{R}+F_{L}-R_{R}-R_{L}-F_{c}\sin\beta \right)\sin\varphi +\frac{1}{m}\left(F_{c}\cos\beta -\mu mg\right)\cos\varphi \right)\Delta t\\ Y_{k}+{\dot{Y}}_{k}\Delta t+\left(\frac{1}{m}\left(F_{R}+F_{L}-R_{R}-R_{L}-F_{c}\sin\beta \right)\sin\varphi +\frac{1}{m}\left(F_{c}\cos\beta -\mu mg\right)\cos\varphi \right)\frac{\Delta t^{2}}{2}\\ {\dot{\varphi }}_{k}+\left(\frac{\left[\left(F_{R}-R_{R}\right)-\left(F_{L}-R_{L}\right)\right]B}{2}\;-M_{r}\right)\frac{\Delta t}{I}\\ \varphi_{k}+{\dot{\varphi }}_{k}\Delta t+\left(\frac{\left[\left(F_{R}-R_{R}\right)-\left(F_{L}-R_{L}\right)\right]B}{2}\;-M_{r}\right)\frac{\Delta t^{2}}{2I} \end{array}\right]$$

### 2.3. RTK-GPS and IMU Fusion Algorithm

During the moderate to high speed turns of the tracked combine harvester, the yaw rate measurement from the IMU sensor will give a bias error that creates a heading drift error, which requires compensation for estimating the absolute heading. For this reason, the model given in Equation (9) was used to estimate the heading of the tracked combine harvester by using a non-linear extended Kalman filter. According to the given Equation (9), the state vector of tracked combine harvester was defined as the Equation (10):

(10) $$X_{k+1}={\left[X_{k+1},Y_{k+1},{\dot{X}}_{k+1},{\dot{Y}}_{k+1},\;{\dot{\varphi }}_{k+1},\;\varphi_{k+1},\varphi_{gk+1},b_{k+1}\right]}^{T}$$

where:

$X_{k+1}$ = Tracked combine harvester position in the east direction at time $t_{k+1}$;
$Y_{k+1}$ = Tracked combine harvester position in the north direction at time $t_{k+1}$;
${\dot{X}}_{k+1}$ = Tracked combine harvester velocity in the X-direction at time $t_{k+1}$;
${\dot{Y}}_{k+1}$ = Tracked combine harvester velocity in the Y-direction at time $t_{k+1}$;
${\dot{\varphi }}_{k+1}$ = Yaw rate at time $t_{k+1}$;
$\varphi_{k+1}$ = Heading of tracked combine harvester at time $t_{k+1}$;
$\varphi_{gk+1}$ = Heading from RTK-GPS at time $t_{k+1}$; and
$b_{k+1}$ = Yaw rate gyro measurement bias at time $t_{k+1}$.

The system measurement vector is defined by the following Equation (11):

(11) $$Z_{k+1}={\left[X_{gps},Y_{gps},\;{\dot{\varphi }}_{imu},\;\varphi_{gps}\right]}^{T}$$

where:

$X_{gps}$ = Measurement position in the east direction from RTK-GPS;
$Y_{gps}$ = Measurement position in the north direction from RTK-GPS;
${\dot{\varphi }}_{imu}$ = Yaw rate gyro measurement from the IMU; and
$\varphi_{gps}$ = Measurement heading from the RTK-GPS.

Now, the extended Kalman filter equations in the following Equations (12) and (13) are considered for the prediction and correction steps; which is maintained in a cyclic manner. The initial state covariance matrix $P_{k}$, process noise covariance matrix $Q_{k}=diag.\left[\begin{array}{cccccccc} 0.002909839 & 0.002909839 & 0 & 0 & 0.000781853 & 0.001350055 & 0.00508 & 0 \end{array}\right]$ and measurement noise covariance matrix $R_{k}=diag.\left[\begin{array}{cccc} 0.00000297878 & 0.00000297878 & 0.000000438461 & 0.010348 \end{array}\right]$ are used for this extended Kalman filter, which were obtained by the static and dynamic test of RTK-GPS and IMU sensors:

*Prediction*:

(12) $$\begin{array}{c} \mathrm{Predicted}\;\mathrm{state},X_{k+1}=f\left(X_{k},u_{k}\right)\\ \mathrm{Predicted}\;\mathrm{covariance},P_{k+1}=A_{k}P_{k}A_{k}^{T}+Q_{k}\\ \mathrm{Predicted}\;\mathrm{measurement},Z_{k+1}=h\left(X_{k+1}\right)\; \end{array}\}$$

*Correction*:

(13) $$\begin{array}{c} \mathrm{Estimate}\;\mathrm{state},{\hat{X}}_{k+1}=X_{k+1}+Kv_{k}\\ \mathrm{Estimate}\;\mathrm{covariance},\;{\hat{P}}_{k+1}=P_{k+1}\left(I-KH_{k}\right) \end{array}\}$$

where:

Innovation, $v_{k}=Z_{k+1}-\;h\left(X_{k+1}\right)$
Kalman gain, $K=P_{k+1}H_{k}^{T}S^{-1}$
Innovation covariance, $S=H_{k}P_{k+1}H_{k}^{T}+R_{k}$

The Jacobian matrices of $A_{k}$ and $H_{k}$ in the prediction step are the following, in which $A_{k}$ was obtained from the partial derivatives of each state vector by using the Equation (9), and $H_{k}$ was obtained from the partial derivatives of each measurement vector.

$$A_{k}=\frac{\partial f}{\partial x}=\left[\begin{array}{ccc} \frac{\partial f_{1}}{\partial x_{1}} & \cdots & \frac{\partial f_{1}}{\partial x_{m}}\\ \vdots & \ddots & \vdots \\ \frac{\partial f_{n}}{\partial x_{1}} & \cdots & \frac{\partial f_{n}}{\partial x_{m}} \end{array}\right]\;\mathrm{and},\;H_{k}=\frac{\partial h}{\partial x}=\left[\begin{array}{ccc} \frac{\partial h_{1}}{\partial x_{1}} & \cdots & \frac{\partial h}{\partial x_{m}}\\ \vdots & \ddots & \vdots \\ \frac{\partial h_{n}}{\partial x_{1}} & \cdots & \frac{\partial h_{n}}{\partial x_{m}} \end{array}\right]$$

## 3. Results and Discussion

### 3.1. Trajectory of Tracked Combine Harvester

**Figure 4:**
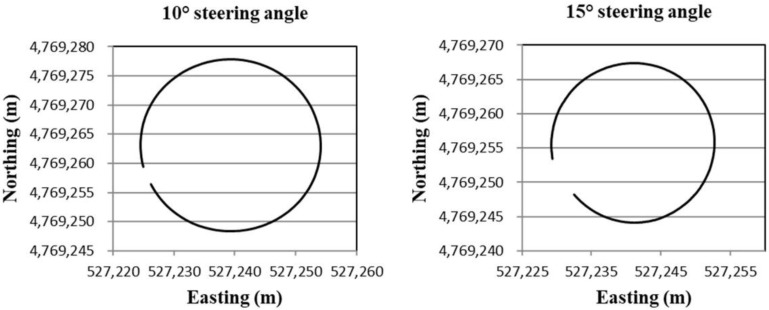
Circular trajectories of the tracked combine harvester at 10° and 15° input steering angles.

**Figure 5:**
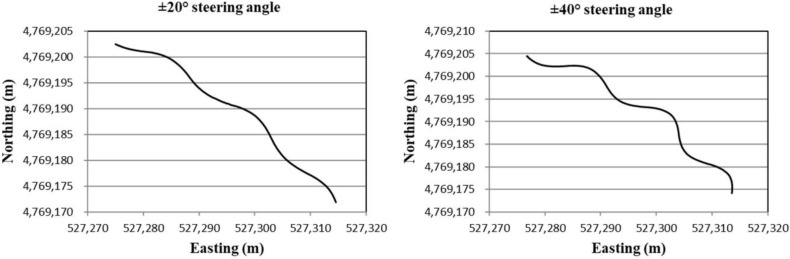
Sinusoidal trajectories of the tracked combine harvester at ±20° and ±40° input steering angles.

For the evaluation of the estimated heading for the tracked combine harvester by compensating the yaw rate gyro measurement bias under non-linear conditions, the tracked combine harvester was run in circular and sinusoidal shapes, and during this time, the sensor measurements were logged, and the running trajectory of the tracked combine harvester can be obtained by using RTK-GPS position. Figure 4 shows the circular trajectories of the tracked combine harvester from the different input steering commands that were at 10° and 15°, respectively. On the other hand, the sinusoidal trajectories of the tracked combine harvester were made by a series of input steering commands such as ±20° and ±40°, respectively, as shown in Figure 5. Based on these circular and sinusoidal trajectory sensor measurements, the absolute heading of the tracked combine harvester was estimated by the extended Kalman filter combined with the tracked combine harvester model.

### 3.2. Estimated Heading of Circular Trajectory

**Figure 6:**
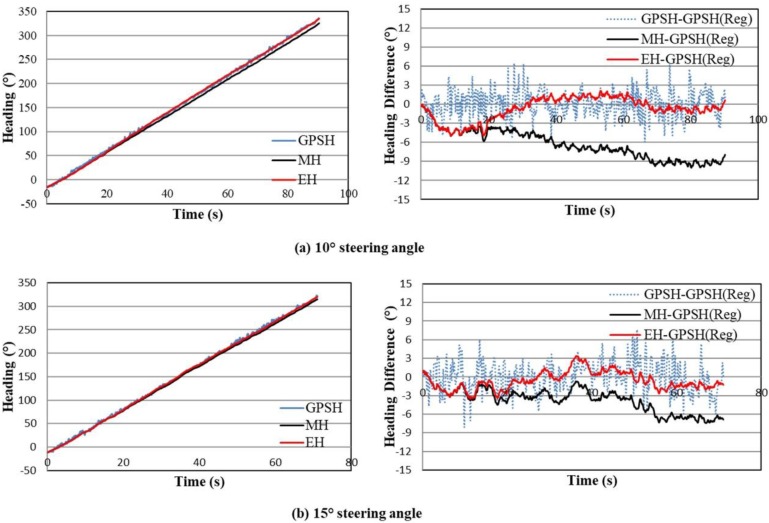
Measured and estimated headings (left figures) for circular trajectories as well as heading difference (right figures) (where, GPSH = GPS Heading, MH = Measured Heading, EH = Estimated Heading and GPSH (Reg) = Linear Regression of GPS Heading).

Figure 6 indicates the measured and estimated heading of circular trajectories (left figures) as well as the difference of these headings from the reference GPS heading (right figures) which was obtained by the linear regression of the noisy trend GPS heading. The heading from GPS measurements is indicated by a blue line, where the measured and estimated headings are marked by the black and red lines. In general, a full circle rotation is counted to 360°, and when the tracked combine harvester was run in the field with a constant steering command, therefore, a full trajectory of circle will be 360°. That why, the left Figure 6a,b indicate that the output headings were bounded by 360°. According to these figures, the red line tried to follow the blue one, which means the estimated heading based on the extended Kalman filter was consistent with the GPS heading. In addition, the heading difference analysis is shown in right Figure 6a,b, which indicates the difference between the measured and estimated headings from the reference GPS heading. Since, the GPS heading provides the exact direction of the vehicle that is computed from the exact position of the tracked combine harvester, the linear regression of the GPS heading was used as a reference GPS heading for the evaluation of estimated headings. The result indicated out that the estimated heading (red line) matches the reference GPS heading for all circular trajectories as shown in Figure 6a,b (right figures) rather than the measured heading. 

From the error analysis of these headings, the RMS errors between the measured or estimated heading and the reference GPS heading were calculated. The RMS errors for the measured heading are 6.74 for 10° steering angle and 4.18 for 15° steering angle, whereas the RMS errors for estimated heading are 1.93 for 10° steering angle and 1.58 for 15° steering angle, respectively. The RMS errors for the estimated heading are lower than the measured heading for all circular trajectories, indicating that the heading drift errors caused from the yaw rate gyro measurement bias are compensated. 

### 3.3. Estimated Heading of Sinusoidal Trajectory

**Figure 7:**
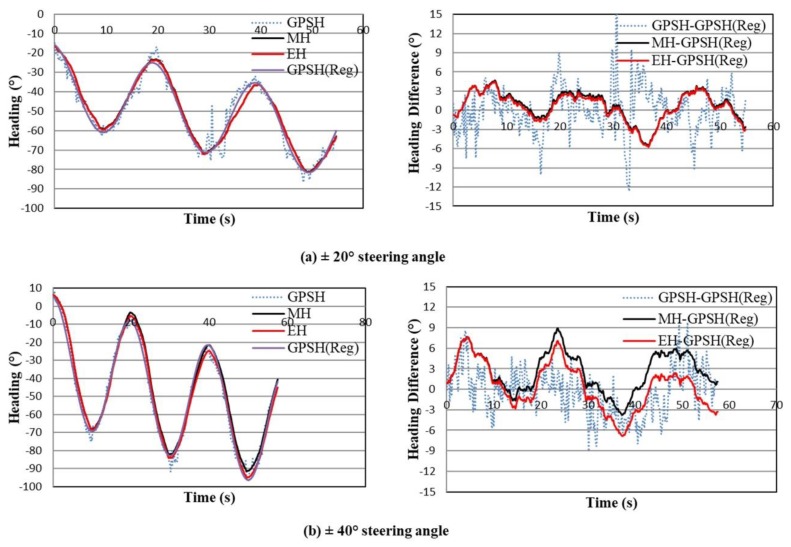
Measured and estimated headings (left figures) for sinusoidal trajectories as well as heading difference (right figures) (where, GPSH = GPS Heading, MH = Measured Heading, EH = Estimated Heading and GPSH (Reg) = Non-linear Regression of GPS Heading).

Figure 7 shows the measured and estimated heading (left figures) and heading difference between the measured or estimated heading and the non-linear regression of the GPS heading (right figures). The purple color line in the left figures indicates the non-linear regression of the GPS heading called the reference GPS heading which was obtained by using the Gauss-Newton algorithm. The Gauss-Newton algorithm is a simple method for solving any non-linear regression problem, and the equation for non-linear regression analysis was considered as $Y_{i}=Asin\left(\frac{X_{i}}{T}+\theta \right)+aX_{i}+b$, where A, T, θ, a and b are the amplitude, period, phase shift angle, phase shift (horizontally) and phase shift (vertically), respectively. On the other hand, the measured and estimated headings were represented by the black and red color lines. According to Figure 7a,b (left side), the estimated heading is better than the measured heading and it tried to match with the reference GPS heading. The heading difference for the estimated heading and measured heading was obtained from the reference GPS heading as shown in the right Figure 7a,b. From the error analysis of measured and estimated heading regarding the reference GPS heading, the RMS errors for the measured heading are 2.39 for ±20° steering angle and 3.96 for ±40° steering angle, whereas the RMS errors for estimated heading are 2.29 for ±20° steering angle and 3.48 for ±40° steering angle, respectively. The RMS errors for the measured and estimated heading do not have a big difference because the reference GPS heading is sometimes matched with the noisy trend GPS heading, and sometimes not matched. If the reference GPS heading matches with the exact GPS heading, the RMS error of measured and estimated heading will change, and the RMS error of the estimated heading will be lower than that of the measured heading. 

### 3.4. Estimated Heading of Concave Polygon Field

**Figure 8:**
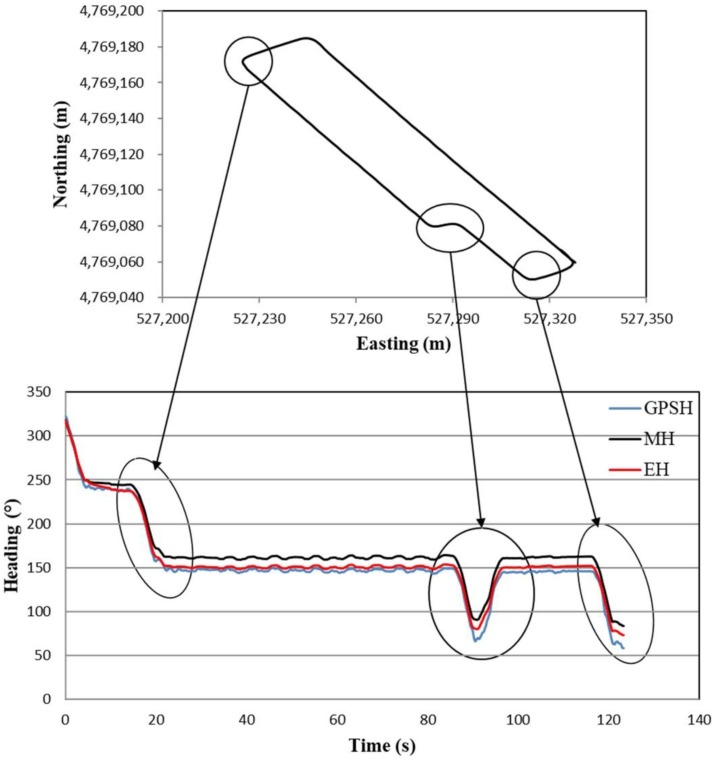
Outlook of a concave polygon field (top figure) and, measured and estimated heading (bottom figure) for the concave polygon field.

**Figure 9:**
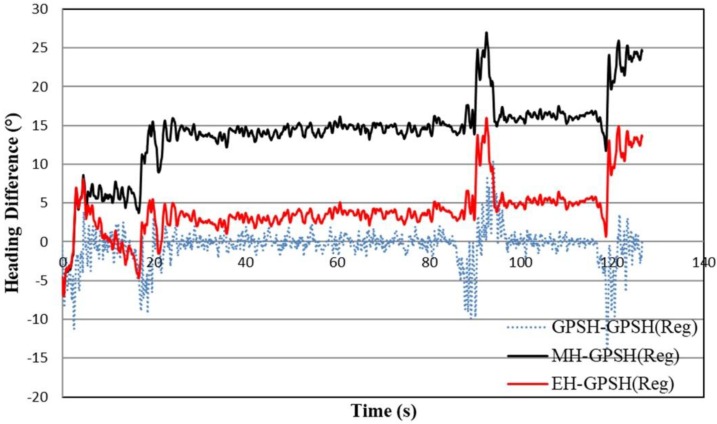
Heading difference based on the reference heading for a concave polygon field.

The heading estimation method based on the tracked combine harvester dynamic model and the extended Kalman filter can also be evaluated for any polygon-shaped field, because this polygon field with crops will be harvested by the robot combine harvester, which is our main research target. Here, only a concave polygon field as shown in Figure 8 was used for this estimation. The circle marked on the concave polygon field (top figure) indicates the turning position of the tracked combine harvester. In these circle positions, the tracked combine harvester cuts the surrounding crop to calculate the exact crop periphery for obtaining the working path of the robot combine harvester by forward and backward movement, and sometimes turns at high speed with low turning radius are considered, which creates a yaw rate gyro measurement bias. This bias creates the measured heading drift error that is important to compensate. Figure 8 (bottom figure) shows a heading of a concave polygon field where the black and red lines are marked for the measured and estimated headings, which was analyzed with respect to the reference GPS heading (blue color line). This reference GPS heading was obtained from the moving average of the noisy trend GPS heading. The estimated heading is better matched with the reference GPS heading than the measured heading in Figure 8 (bottom figure). In addition, the heading difference between the measured heading or estimated heading and the reference GPS heading was calculated, as shown in Figure 9. The RMS error was calculated from the measured or estimated heading and the reference GPS heading, and from the error analysis, the RMS errors for measured and estimating heading were 14.93° and 5.17°, respectively. Since, the RMS error of estimated heading is lower than the measured heading, the estimated heading shows more consistency with the reference GPS heading. The results also describe that the measured heading drift error was compensated by the extended Kalman filter and the tracked combine harvester dynamic model. 

## 4. Conclusions

This research deals with the tracked combine harvester dynamic model, sensor measurements and the extended Kalman filter, which are used to estimate the absolute heading of a tracked combine harvester under non-linear condition by compensating the IMU yaw rate gyro measurement bias which influences the heading drift errors. Different sets of experiments such as circular, sinusoidal and concave polygon trajectories were executed to evaluate this estimation method. The results for different trajectories revealed that the IMU yaw rate gyro measurement bias was compensated based on the extended Kalman filter and the tracked combine harvester dynamic model, and the absolute heading was simultaneously determined, which is better than the measured heading. The RMS errors of the estimated heading are lower than those of the measured heading for all trajectories. Therefore, this estimation method can be used to estimate the absolute heading when the tracked combine harvester will makes turn at high speed and with high order steering commands in order to cut wheat and paddy rice near headlands to calculate the exact crop periphery for the development of a harvesting map, for more details see [25].
